# Advances in Monte Carlo Method for Simulating the Electrical Percolation Behavior of Conductive Polymer Composites with a Carbon-Based Filling

**DOI:** 10.3390/polym16040545

**Published:** 2024-02-18

**Authors:** Zhe Zhang, Liang Hu, Rui Wang, Shujie Zhang, Lisong Fu, Mengxuan Li, Qi Xiao

**Affiliations:** 1 School of Textile Science and Engineering, Tiangong University, Tianjin 300387, China; 16622902533@163.com (Z.Z.); fls147abc@163.com (L.F.); 2 School of Physical Science and Technology, Tiangong University, Tianjin 300387, China; h4ever2022@163.com; 3 College of Fine Arts & Design, Tianjin Normal University, Tianjin 300387, China; mxli@tjnu.edu.cn; 4 School of Textile Garment and Design, Changshu Institute of Technology, Changshu 215500, China; xiaoqi223638@163.com

**Keywords:** carbon-based materials, CPCs, Monte Carlo simulations, electrical percolation behavior, conductive networks

## Abstract

Conductive polymer composites (CPCs) filled with carbon-based materials are widely used in the fields of antistatic, electromagnetic interference shielding, and wearable electronic devices. The conductivity of CPCs with a carbon-based filling is reflected by their electrical percolation behavior and is the focus of research in this field. Compared to experimental methods, Monte Carlo simulations can predict the conductivity and analyze the factors affecting the conductivity from a microscopic perspective, which greatly reduces the number of experiments and provides a basis for structural design of conductive polymers. This review focuses on Monte Carlo models of CPCs with a carbon-based filling. First, the theoretical basis of the model’s construction is introduced, and a Monte Carlo simulation of the electrical percolation behaviors of spherical-, rod-, disk-, and hybridfilled polymers and the analysis of the factors influencing the electrical percolation behavior from a microscopic point of view are summarized. In addition, the paper summarizes the progress of polymer piezoresistive models and polymer foaming structure models that are more relevant to practical applications; finally, we discuss the shortcomings and future research trends of existing Monte Carlo models of CPCs with carbon-based fillings.

## 1. Introduction

Polymer materials are widely preferred in many fields due to the abundance of their raw materials, low cost, ease of processing and preparation, low density, and corrosion resistance [1,2]. However, common polymer materials, such as polyethylene (PE), polypropylene (PP), polyurethane (PU), polyimide (PI), and polyether ether ketone (PEEK), are electrically insulating [3,4,5], which makes it difficult to use them directly in areas that require a high electrical conductivity of the materials, such as electromagnetic interference shielding, aerospace, energy storage devices, and wearable electronics [4,6,7]. Therefore, polymers can be modified by physical or chemical methods to improve their conductivity. Among them, filling polymers with conductive materials to prepare conductive polymer composites is the most efficient and convenient method, which can improve the conductivity of polymers under the assumption of guaranteeing other use indicators [8]. Conductive materials can be classified into intrinsic and filled types according to the type of structure [9]. Intrinsic-type conductive materials include polyacetylene, polyaniline, and polypyrene. Most intrinsic-type conductive materials have relatively low electrical conductivity (<100 S/cm), and the preparation of intrinsic-type conductive materials with high electrical conductivity is technically and technologically difficult [10,11,12]. Compared with intrinsically conductive materials, filled conductive materials have excellent electrical conductivity, either by interconnecting within the polymer or by tunneling, in the form of electron transfer [13,14]. Both of these conductive carbon materials, compared with conductive metal and metal oxide materials, have excellent electrical conductivity and chemical stability, and the material density is low and light, and the raw material is cheap and easy to obtain, so they have been widely used in the field to improve the conductivity of polymers [9,15,16]. The structure and properties of commonly used carbon conductive fillers are shown in Table 1.

Electrical percolation behavior is one of the main concerns regarding carbon-based fillings in CPCs, and the percolation threshold is defined as the critical filler fraction of the polymer when it transitions from an insulating to a conductive state [20,21,22,23]. Conventional experimental methods often require a large amount of time and material to achieve the percolation threshold and are very limited at the microscopic level in terms of providing a better understanding of the changes in the conductive network in the polymer system. In contrast to experimental methods, a computer simulation is a very important and effective method, which can not only derive the percolation threshold of conductive materials in polymer systems using models and equations but also provides an in-depth study and analysis of the changes in conductive networks in filled polymer systems from a microscopic point of view [24]. Therefore, in recent years, numerous studies have reported the modeling of carbon-based fillings in conductive polymer composites constructed by different simulation methods. For example, Rahaman et al. [25] applied different sigmoidal models (S-models) to predict the percolation thresholds of conductive composites filled with CB and CNF and compared them with the percolation thresholds of classical percolation theory, as well as with the results in the published literature. It was found that all models, except the Sigmoidal-Logistic-1 (SL-1) model, can be successfully used to determine the percolation thresholds of externally conducting polymer composites. Feng et al. [26] developed a micromechanical model to predict the percolation threshold of CNT–polymer nanocomposite systems and investigated the effect of the size of CNTs on the percolation behavior of the composites. Qu et al. [27] used molecular dynamics simulations to study the probability of the conductivity of polymer nanocomposites with mixed ball and rod fillers in static and shear fields and analyzed the synergistic conductivity effect between ball and rod fillers. Wang et al. [28] studied the conductivity of CB–polymer composites based on simulations of the finite-volume method (FVM) and discrete element method (DEM), through which it was possible to predict the amount of CB required to achieve the target conductivity of the polymer composites. All of the above simulations have been shown to be able to predict the electrical percolation behavior of filled polymers. However, these models also suffer because of the complexity of the algorithms and the lack of stochasticity in the model for the insertion of conductive materials into polymers.

The Monte Carlo algorithm originated from Buffo’s circumference measurement by the needle-throwing method. This algorithm is simple and can simulate a random body image constructed by randomly inserting, moving, deleting, and rotating indeterminate states of particles under certain system conditions, and it can solve the problems of the above simulation, and it is the most commonly used method to simulate the electrical percolation behavior of filled conductive polymers at present. In the 1970s, Pike and Seager [29] were the first to use the Monte Carlo method to simulate polymer systems filled with spherical-, square-, and rod-shaped materials, and they investigated the critical size of the particles when they reached the percolation state by adopting the inclusive figure (IF) method, which only changes the size of the particles. On this basis, subsequent researchers carried out more in-depth and systematic studies, which gradually improved the Monte Carlo model of filled conductive polymers. In the 1980s, Balberg [30] discussed the percolation behavior of interpenetrating structural cylindrical packings using the overlap figure (OLF) method with constantly changing numbers of particle and further investigated the effects of the aspect ratio and orientation of the packing on the percolation behavior [31]; this study was the earliest to simulate the percolation behavior of electrically conductive materials in three-dimensional space using the Monte Carlo method with the overlap figure (OLF), creating a precedent in this field. In the 1990s, Louis and Gokhale [32] studied the percolation behavior of spherical fillers in 3D space, considering no interpenetration and overlap of the conducting materials inside the actual polymers and setting the outermost 10% of the radius from the center of the filler sphere as the permissible overlap range of the two spheres. They also set the distance between the centers of the two filler spheres to be less than the sum of the radii of the two spheres and more than 90% of the sum of the radii as the condition for determining whether or not the two spheres are connected. On the basis of the above settings, the formation of conductive pathways in the system was determined, and the percolation threshold of the system was simulated. On this basis, Li and Kim [33] applied the theory of the tunnel effect in the electrical conductivity mechanism to the three-dimensional Monte Carlo simulation of graphitic materials filled with polymers and set the tunneling distance to be 10 nm; that is, the two fillers were judged to be connected when the spacing of the outer edges of the two fillers was less than 10 nm, and with this setting, the effects of the variations in the intergraphitic distances, graphite diameters, and thicknesses on the percolation behaviors were investigated. Additionally, Toshiaki et al. [34] proposed a modified model of a finite-width stick and investigated the effects of the L/D ratio and model orientation on the percolation threshold, finding that a model L/D ratio greater than 40 or an orientation angle of less than 30 degrees had a greater effect on the percolation threshold. This paper outlines the early developments in the use of Monte Carlo methods to simulate the electrical percolation behavior of filled polymer composites. To this end, a preliminary modeling system for polymers filled with conductive materials was developed. Recent work in this area is summarized in more detail in Section 3.

Scholars have reviewed various aspects of the use of carbon-based fillings in CPCs, Khan et al. [5] reviewed the methods used to achieve low/ultralow electrical percolation thresholds in carbon-filled PP nanocomposites. Faridirad [35] and Goncalves et al. [36] reviewed the use of different carbon-based materials in polyamides and polylactic acid matrices, respectively, for the production of low-resistivity polymer composites. Tripathi [37], Mohan [38], and Kausar et al. [39] reviewed the application of graphene and its derivatives in polymer matrices. However, no review has yet summarized the simulation of the electrical percolation of carbon-based fillings in CPCs. Therefore, this paper takes the Monte Carlo model of carbon-filled CPCs as the research object. First, the theoretical basis of the model is introduced, including the conductive percolation phenomenon and the conductive mechanism of filled polymers. The research progress in this field over the past two decades is summarized, and the construction method of the model and the influencing factors of the polymer’s conductive properties analyzed by the model are discussed in detail from the perspectives of the shape and structure of the carbon-based materials and the integration with practical applications, respectively. Finally, the shortcomings of the existing research in this field and future research trends are discussed.

## 2. Filled Polymers’ Conductive Percolation Phenomenon and the Theory of Conductive Percolation Model Construction

The behavior of polymer composites is affected by several factors, including the parameters of the conducting material and its interaction with the polymer matrix. To more realistically model the conductive percolation of filled polymer composites, it is necessary to fully understand the phenomenon of electrical percolation and theory related to constructing electrical percolation models. These two parts are described below.

### 2.1. Filled Polymers’ Conductive Percolation Phenomenon

**Figure 1:**
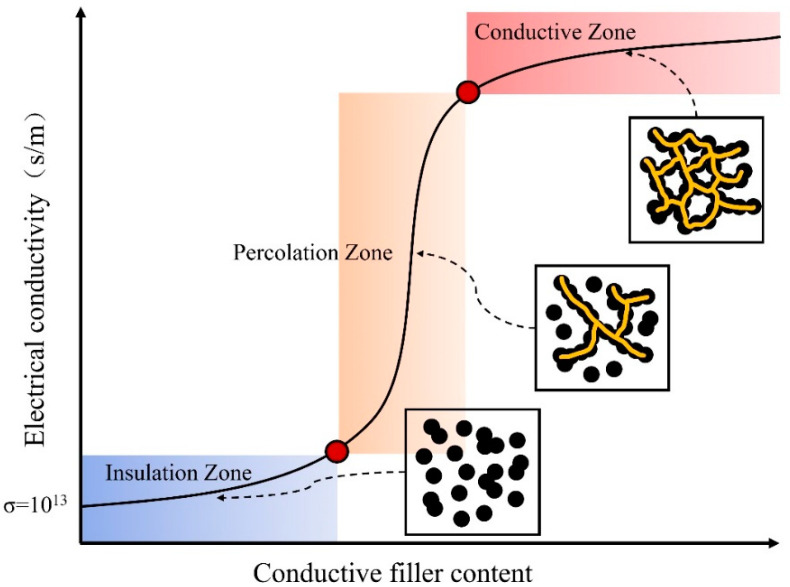
Conductivity of filled CPCs vs. addition of conductive materials.

Most polymer materials have been found to have a resistivity of between 10^12^ and 10^17^ Ω·cm [3], and the addition of conductive fillers, such as CB, CNTs, and graphene, can make polymers electrically conductive; a large number of experiments and studies have demonstrated that the conductivity of polymer composites has a nonlinear relationship with the doping of conductive materials. In Figure 1, there exists a threshold value for doping of the conductive material. When the conductive filler doping is below the threshold, the conductivity of the composite material increases slowly with the increase in filler doping. However, when the doping of the conductive filler is close to the threshold, the conductivity of the material increases dramatically and changes from an insulating material to a conductive material. Subsequently, when the doping of the conductive material is further increased with yet another specific value, the electrical resistivity of the polymer composite material tends to stabilize again, and a further increase no longer significantly improves the resistivity of the material [40]. This phenomenon is known as the electrical percolation of filled polymers, and the amount of conductive material doped at the time of a sudden change in conductivity is known as the percolation threshold of the polymer composite [41].

### 2.2. Theory on the Construction of a Conductive Percolation Model for Filled Polymers

The theory behind constructing a conductive percolation model for carbon-based fillings in conductive polymer composites is based on the conductive mechanism of the conductive material in the polymer system. The way carriers move in polymers and conductive materials determines the conductive mechanism of polymer composites. Currently, the three main carrier migration mechanisms are: macroscopic percolation theory, tunneling effect theory, and field emission theory [42,43,44].

The macroscopic percolation theory is also called conductive channel theory. According to this theory, when the number of conductive materials in the filling is low, the conductive materials are separated from each other by the polymer matrix; along with an increase, to a certain level, in the number of conductive materials, the conductive materials connect to each other in the polymer matrix to form a conductive network for electron transport, and the resistivity of the polymer is, therefore, significantly reduced. With the continued addition of conductive materials, the electron transport channels in the polymer matrix become saturated, and the corresponding resistivity no longer changes significantly [45]. After the polymer material undergoes percolation ($p>p_{c}$), the relationship between its electrical conductivity and the filler’s own conductivity, the percolation threshold, and the volume fraction is usually expressed by the following equations [43,46]:

(1) $$\sigma =\delta {\left(p-p_{c}\right)}^{t}$$

where σ is the polymer composite’s conductivity, δ is the conductivity-related constant of the conducting material, p is the mass fraction of the conducting material, $p_{c}$ is the polymer composite percolation threshold, and t is the conductive network dimensionality critical factor.

In 1957, Polley and Boonstra’s study found that a sudden change in electrical resistivity occurred in silicone rubber (SR) before the fill level of the CB particles reached the theoretical value required to construct a conductive network, so it was envisaged that conductive materials would be sufficient to form a channel for electron transport in a polymer matrix without complete contact [47], which is the theory of the tunneling effect. Tunneling theory suggests that the formation of a conductive network does not depend on direct contact among the conducting materials, but that when the conducting materials are close enough to each other, the electrons can acquire thermal energy from the surrounding environment, thus overcoming the potential barrier imposed by the polymer and allowing the electrons to leapfrog, generating an electron tunneling current [48,49,50,51].

Simmons et al. [52] further analyzed the theory of the tunneling effect and derived the formula for the calculation of the tunneling effect with an arbitrarily shaped potential barrier, as shown in Equations (2)–(4), as follows:

(2) $$J=J_{0}\left\{\bar{\varphi }exp\left(-A\varphi^{-\frac{1}{2}}\right)-\left(\bar{\varphi }+eV\right)exp{\left[-A\left(\bar{\varphi }+eV\right)\right]}^{\frac{1}{2}}\right\}$$

(3) $$J_{0}=\frac{e}{2\pi h{\left(\beta ∆s\right)}^{2}}$$

(4) $$A=\left(\frac{4\pi \beta ∆s}{h}\right){\left(2m\right)}^{\frac{1}{2}}$$

where e is the electron charge, h is Planck’s constant, Δs represents the difference between the potential barriers at the Fermi energy level, φ is the average barrier height, and V is the voltage on both sides.

In addition, the Landauer–Büttiker formula [53] for contact junction tunneling in electron transport vis ballistic trajectories can also be used to calculate the tunneling resistance among conducting materials:

(5) $$R_{contact}=\frac{h}{{2e}^{2}}\times \frac{1}{M\left[T+\frac{\pi }{6}{\left(k_{B}t\right)}^{2}\frac{d^{2}t}{dE^{2}}\right]}$$

where e is the electron charge, h is the Planck’s constant, M is the total number of conducting channels in the filler–polymer system, T is the transport probability, t is the temperature of the system, $k_{B}$ is Boltzmann’s constant, and E is the electron energy level.

Tunneling theory suggests that the volume fraction of the conductive material in a polymer system affects its dispersion, as well as the distance between particles of the material, which, in turn, affects the conductivity of the polymer system. When the volume fraction of the conductive material is less than a certain critical value, the distance between the filler particles cannot reach the shortest distance for the tunneling effect to occur; therefore, the electron transfer between particles cannot occur. So, only the volume fraction of the conductive material in a fixed range can be applied to tunnel effect theory.

Additionally, researchers have found that at high temperatures or high voltages in the external environment, the phenomenon of conductivity also occurs when the number of conductive materials added to the polymer is small, that is, the field-emission effect. Field emission theory suggests that, under the action of a strong external environment, conductive particles collide with each other more frequently, thus generating electric currents to form an emissive electric field such that electrons can overcome the binding of the nucleus in the conductive material during free movement in the formation of electric currents [54]. Generally, the field emission effect occurs in polymer composites only when there is localized overheating and high voltage [55].

Currently, the mainstream view of the conductive theory of filled CPCs is that three conductive mechanisms coexist. According to the above conductive mechanism, in the carbon-based fillings in CPCs of the electrical percolation model, when the model considers the applied electric field and the filler concentration is low, there is a high probability that the conductive material between the emission of the electric field, at this time, the field emission mechanism occupies a dominant position. When the volume fraction of the conductive material in the model increases to the point where the tunneling distance between particles is reached, a tunneling current is generated between the particles of the conductive material and the tunneling mechanism dominates. With the further increase in the volume fraction of the conductive material in the model, the direct contact between the conductive material forms a conductive network, and the macroscopic percolation theory dominates at this time. In the simulation process, the corresponding conductive mechanism is applied according to the condition parameters of the model.

## 3. Monte Carlo Models for the Electrical Percolation Behavior of Polymers with a Carbon-Based Filling

The Monte Carlo method, as a continuous method for generating random bodies of images, can accurately simulate the random distribution of fillers in space. Therefore, it is well suited for modeling filled polymer composite systems. The Monte Carlo method is used to simulate the electrical percolation behavior of carbon-based fillings in conductive polymer composites as follows: According to the distribution characteristics of the actual conductive material in the polymer system, the simulated particles of conductive material are randomly placed into a volume unit. The mutual contact between particles will form a conductive path in space, and based on the probabilistic model of the formation of the conductive path, the percolation threshold of the conductive material in the polymer system is derived by solving the average value of a large number of different configurations under the same configuration parameters [51]. The models also allow for a microscopic analysis of the influence of the conductive materials, as well as the polymer’s parameters, on the electrical percolation behavior. In this section, we summarize the progress on the Monte Carlo modeling of rod-shaped CNTs, spherical CB, plate-like graphene materials, and hybrid materials for use in filling polymers.

### 3.1. Monte Carlo Models of CNT-Filled Polymers

Carbon nanotubes (CNTs) have ultrahigh aspect ratios and excellent electrical properties, and the conductive percolation behavior of CNTs can occur at low filling levels in polymers, making them ideal conductive fillers in CPCs [56,57,58]. In recent years, use of the Monte Carlo method to simulate the conductive percolation behavior of CNT-filled polymers has attracted much attention, and scholars have established different Monte Carlo models for CNT-filled polymers according to their own research content. Of these, they are classified according to the dimensionality of the distribution space of the models, including 2D models and 3D models.

**Figure 2:**
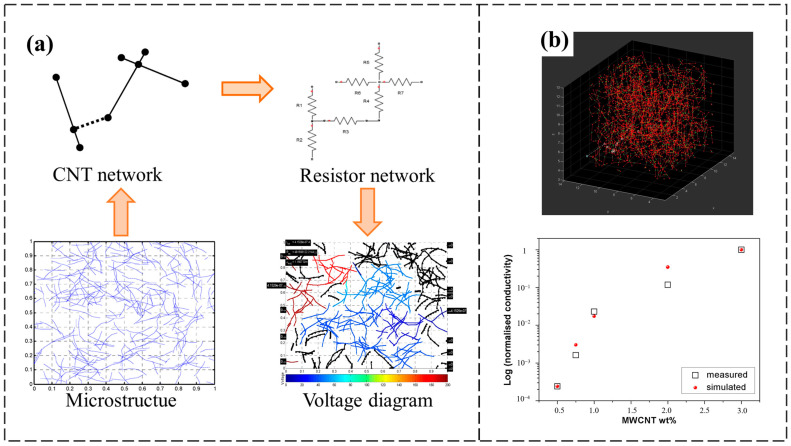
(**a**) Schematic diagram of the equivalent resistance calculation for the 2D model [60]; (**b**) cloud diagram of the CNT-filled RVE with the model and experimental conductivity results [61].

Two-dimensional models are often used to simulate the electrical percolation behavior of CNTs in polymer films. Zeng et al. [59] developed a 2D Monte Carlo model of MWCNTs dispersed as one-dimensional rods and narrow two-dimensional rectangles in a representative volume element unit RVE (hereafter collectively referred to as RVE). It was found that the prediction of the percolation threshold of the narrow two-dimensional rectangular model was closer to the experimental results because it took into account the L/D ratio of the CNTs. Although the 2D model could predict the percolation threshold of MWCNTs in epoxy resin more accurately, the electrical conductivity of the model was not calculated. Soto et al. [60] developed a 2D model to predict the electrical percolation behavior of CNT-filled polymers. As shown in Figure 2a, in RVE, interconnected CNTs are identified by incorporating the tunneling effect and constructed as an equivalent circuit, and the tunneling resistance between CNTs was calculated according to the Simmons [52] formula. The equivalent resistance network was transformed into a system of linear equations using nodal voltage analysis, and, finally, the equivalent resistance and conductivity of the model were obtained by solving with the Gauss–Jordan elimination method. The resistance predicted by the model formed a good fit with the experimental results for the thin film polymers.

Compared with the 2D Monte Carlo model, the 3D model considers the more complex spatial distribution of CNTs in the RVE space and more truly reflects the spatial distribution of CNTs inside the polymer. In the 3D Monte Carlo model, CNTs can be regarded as single or multilayer coaxial hollow tubes composed of hexagonally arranged carbon atoms, which are often constructed as straight rod-like structures for convenience. Micaela et al. [61] established a 3D Monte Carlo model of a CNT-filled epoxy resin using Matlab and used Dijkstra’s algorithm to find the shortest conductive paths for the CNTs between two boundaries of the polymer, thus determining the conductive network in the model. A comparison of the models’ simulation results with the experimental data are shown in Figure 2b, which proves that there is a good correspondence between the model and the experimental results. On this basis, Stelmashchuk et al. [62] compared the hard-core model and the soft-core model of CNTs by considering the interpenetration and overlap of CNTs in the matrix. The outer part of the hard-core model can be penetrated, while the inner part cannot. The outer region also serves as a tunneling effect region between the CNTs. The soft-core model has CNTs that can penetrate each other. The simulation results of the hard- and soft-core models are shown to have the same trend, but there is a difference in the amount of carbon tubes added to reach a certain resistance, with the hard-core model being more consistent with the experimental results. Tang et al. [63] used Matlab software to build a hard-core model of CNTs and designed an efficient network search method for conductive networks. The method searches for CNTs that may have contact near other CNTs using the KD-tree algorithm; then, it analyzes the contact among the CNTs, searches for a complete conductive network, and, finally, calculates the conductivity of the equivalent resistive network according to Kirchhoff’s law of resistance. An analysis of the model’s parameters led to the following results: neglecting the intrinsic resistance of CNTs had little effect on the calculation of the equivalent resistance of the conductive network; the percolation threshold is related to the morphology of the CNTs, and the L/D ratio will be smaller. Among the above studies on a straight rod-like structure CNT model, the CNT models were all set to be randomly distributed in the RVE. Matos et al. [64] took into account the distribution of the CNTs in the polymer, used microscopy and dispersion processing to derive the probability density function of the concentration of CNTs, and used Abaqus software to construct a model of a nonuniformly distributed CNT-filled polymer. In this simulation, the nonhomogeneous distribution of the CNTs was 11% smaller than the conductivity of the uniform distribution, and the nonhomogeneously distributed CNT model was closer to the actual experimental situation.

**Figure 3:**
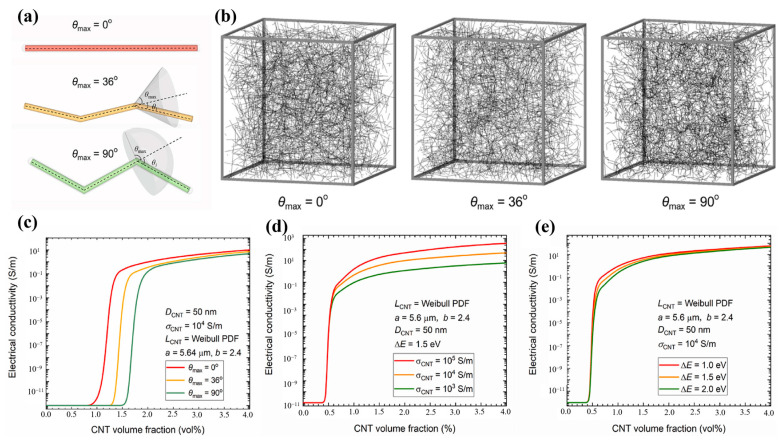
(**a**) Schematic diagram of waviness in the CNT model; (**b**) schematic of the CNT-filled RVE with different waviness degrees; (**c**–**e**) effect of model parameters on the polymer’s conductivity; (**c**) CNTs’ waviness; (**d**) CNTs’ intrinsic conductivity; (**e**) polymer potential barrier [68].

In fact, CNTs are not straight rods in the polymer matrix but rather curved segments that are intertwined with each other. Therefore, modeling CNTs as curved or folded structures can more realistically simulate the shape and structure in polymers. Taking into account the actual structure of CNTs in polymers and the simulation efficiency, Sun et al. [65] developed a CNT model with a maximum folding angle of 120°. The model uses Dijkstra’s algorithm to search the conductive network, calculates the tunneling resistance between CNTs according to the Landauer–Büttiker formula, and finally applies Kirchhoff’s law of resistance to solve the model conductivity. The model uses Dijkstra’s algorithm to search the conductive network, calculates the tunneling resistance between CNTs according to the Landauer–Büttiker formula, and finally applies Kirchhoff’s law of resistance to solve the model conductivity. The researchers verified the accuracy of the model by comparing the simulation results with the experimental results; moreover, the analysis of the model study reveals that the polymer conductivity increases with the increase in the L/D ratio of the CNTs. The effect of the degree of the CNTs’ agglomeration on the polymer conductivity changes with the change in the volume fraction of the CNTs; at lower volume fractions, the conductivity increases with an increase in the degree of agglomeration and then decreases after reaching the maximum value; at higher volume fractions, the conductivity decreases monotonically with an increase in the degree of agglomeration. Fang et al. [66] established a 3D curved CNT model based on stochastic third-order Bézier curves and investigated the effect of the degree of the CNTs’ curving on the electrical conductivity of the model. The results show that the conductivity decreases significantly with an increase in the degree of Bézier curvature, and the percolation threshold of the curved CNT model increased by approximately 20% compared with that for the straight CNTs. Doh et al. [67] investigated the effect of the degree of waviness of CNTs on the conductivity and the chance of percolation using a Monte Carlo method based on Bayesian inference. The percolation behavior of the polymer was simulated by increasing the maximum polar angle of the CNTs, and the final simulation results were in agreement with those of Fang [66]. The inhomogeneity and waviness of the length of CNTs, which are inevitably present in real CNT-filled polymers, are the two main factors that affect the electrical percolation behavior of polymers. However, these two factors have rarely been considered simultaneously in the previous models. Wang et al. [68], on the basis of an existing model and considering both the length inhomogeneity and the waviness of CNTs, developed a new numerical model (Figure 3a,b). The model describes the length inhomogeneity of CNTs in terms of the Weibull distribution, and a segmented sequence of several line segments was used to simulate the curved CNTs. It was found that the polymer’s electrical percolation behavior depended mainly on the geometry and shape of the filler rather than the intrinsic conductivity of the filler or the potential barrier of the polymer; the percolation threshold decreased significantly as the length inhomogeneity of the CNTs increased or the degree of waviness decreased. Specific results are shown in Figure 3c–e.

In this section, representative research advances in the Monte Carlo method for simulating the electrical percolation behavior of CNT-filled polymers are summarized in terms of the spatial dimensions of the model and the shape and structure of the CNTs. It can be seen that the research in this field is relatively mature. In addition to the studies presented above, many other researchers have also achieved impressive results in the Monte Carlo simulation of CNT-filled polymers, which are summarized in Table 2. Moreover, we also compiled the results of Monte Carlo simulation and experiments in the above research and put them in Table A1.

### 3.2. Monte Carlo Models of CB-Filled Polymers

Carbon black (CB) is the material of choice for industrialized conductive fillers due to its mature production process, stable performance, and low price [90]. The microstructure of CB-filled polymer composites is very complex, and its percolation behavior is affected by a variety of factors, which cannot be experimentally analyzed. Therefore, researchers constructed a Monte Carlo model for CB-filled polymer conductive composites, that successfully predicts the electrical conductivity of CB-filled polymers, and analyzes the influencing factors of the polymer’s percolation behavior from a microscopic point of view. The latest research progress in this field is summarized below.

CB particles are spherical or subspherical in shape, with diameters ranging from tens to hundreds of nanometers, and because of the special physical and chemical properties of CB, they are not dispersed in polymers as individual particles but as aggregates. In general, low-structured aggregates consist of 30–100 CB particles, and high-structured aggregates contain 100–300 CB particles [91,92]. On the basis of the shape–structure analysis of CB in polymers, its aggregates are used in models instead of single CB particles as the basic unit of the conductive phase, which is often modeled as an equivalent sphere containing both CB particles and polymer matrix because CB aggregates do not have a regular shape.

**Figure 4:**
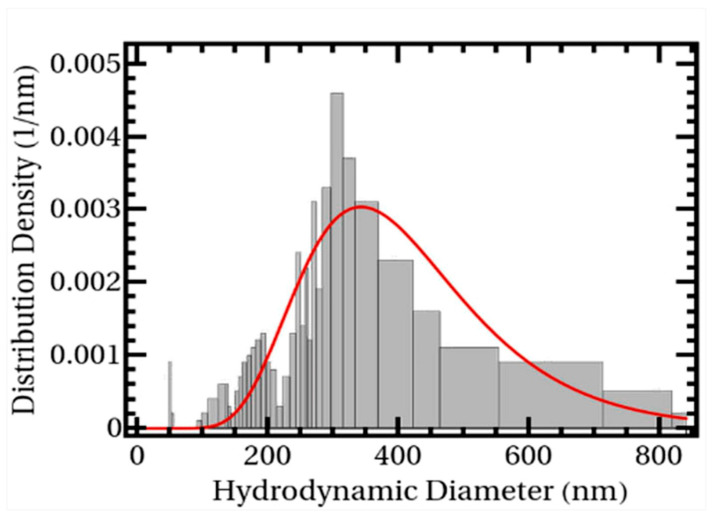
Size distribution of the CB particles [96].

Vas et al. [93] studied the percolation behavior of spherical fillers in SR. Among them, the formula for contact resistance was derived from the Landauer–Büttiker formula, and then an equivalent conductivity network was established from Kirchhoff’s current law to calculate the model final conductivity, and the model predictions were in good agreement with the experimental results. This method can be easily applied to any other filler-polymer combination. It can also be used as a predictive tool for designing simple composites with desired electrical properties. Arun et al. [94] developed a Monte Carlo model for CB-filled PU based on the Visual “C” algorithm. The model determines whether percolation behavior occurs by calculating the probability of the connectivity of 100 randomly distributed conformations of any volume fraction of CB in RVE. The simulation results show that a percolation probability greater than 50% is observed for a mass fraction of 6.2% CB in RVE, which is in agreement with the experimental test results of a percolation threshold of 6%. Ji et al. [95] proposed a calculation method for the electrical conductivity of CB-filled polymers based on the equivalent resistance algorithm of large-scale pure resistance networks. Through a comparison of the model and the experimental results, it was verified that it is feasible to obtain the conductivity of polymer composites directly by the equivalent resistance calculation method of the large-scale pure resistance network. For convenience, the above studies equated the CB agglomerates to a sphere model with the same particle size, but this does not correspond to the actual distribution of CB in the polymer matrix. Coupette et al. [96] considered this problem and investigated the effect of the particle size distribution of the CB agglomerates on the percolation behavior using the Monte Carlo model, where the particle sizes of the CB conformed to a lognormal distribution (Figure 4). It was found that an increase in the average particle size of CB aggregates reduced the percolation threshold, but the particle size distribution function had no effect on the percolation threshold.

The abovementioned research focuses on the prediction of percolation thresholds by the model and the comparison with the experimental results. In addition to this, researchers have also carried out studies on the factors that influence the model. Zhu et al. [97] researched the adaptability of the conductive filler to the geometry and size of the RVE in a Monte Carlo model. This study showed that it is reasonable to use RVE for simulation, and the size of RVE can be determined based on the chi-square test results of the value of percolation threshold, suggesting that the cube is used as the standard shape of the RVE. In the Monte Carlo model, the method of searching for conducting paths affects the computation time of the model and may also affect the calculation of the final conductivity of the model. Bozyel et al. [98] performed simulations using the Python programming language and Visual Studio-Code to determine the percolation thresholds of CB-filled polymers in a Monte Carlo model of CB-filled polymers by means of a pathfinding algorithm, as shown in Table 3, comparing pathfinding algorithms, such as A*, A* depth, best-first search, breadth-first search, and Dijkstra, and analyzing the speed of computation of the algorithms, the conductivity pattern defined when visiting the nodes and the calculated percolation threshold results. The electrical properties of TPU and PVA polymers filled with different ratios of CB were investigated experimentally and then compared with the simulation results. All of the algorithms’ predicted results were in good agreement with the experimental results, among which, the best-first search algorithm was the fastest in the simulation, with a simulation time of 2.288 s. Mazaheri et al. [99] used a hard-core CB model to investigate the effects of the electron tunneling effect, the polymer potential barrier, the interfacial thickness, the packing radius, and other factors on the percolation behavior. The results show that for every 1 nm decrease in the tunneling distance, the barrier height decreased by 1 eV, and the effective conductivity increased by more than an order of magnitude in all cases. The decrease in packing radius and the increase in interfacial thickness positively affect the percolation behavior.

### 3.3. Monte Carlo Models of Graphene-Filled Polymers

The conductivity of graphene can be as high as 10^2^–10^4^ S/cm, and the large specific surface area makes it easier to construct long-range, through-conducting network structures in polymer matrices, so graphene has been widely used to prepare CPCs [100,101,102,103,104]. Limited by the inability to obtain microstructural information within CPCs by experimental means [105,106], a series of studies on the Monte Carlo modeling of graphene-filled CPCs has been carried out in recent years, which is systematically summarized in this section.

**Figure 5:**
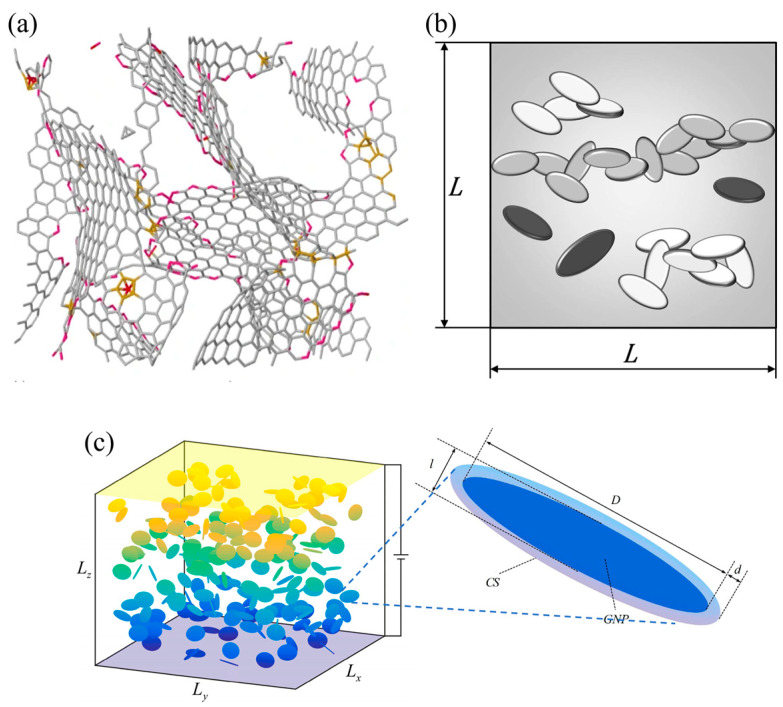
(**a**) 3D graphene network model [107]; (**b**) schematic of the disk-shaped graphene model filled with RVEs, with individual graphene in black, aggregated graphene in white, and percolation network in grey [108]; (**c**) schematic of the graphene model for coated surfaces (CSs) [110].

In polymer systems, graphene fillers tend to exist in the form of lamellar stacks with irregular polygonal shapes along with some folds. Mathioudakis et al. [107] constructed a real graphene model using the Monte Carlo method to simulate and theoretically investigate the structural, mechanical, and optoelectronic properties of three-dimensional graphene fillers. As shown in Figure 5a, although all of the properties are closer to the real graphene material, the model is too complicated and is not favored by researchers. In contrast, researchers tend to approximate graphene models as simpler disks or rectangular plates. In Figure 5b, Oskouyi et al. [108] constructed a three-dimensional Monte Carlo model by modeling graphene as a disk added to the RVE and investigated the effect of an external electric field voltage on the electrical conductivity of the polymer composite. The results showed that the resistivity of the polymer composites decreased nonlinearly with the increase in the applied voltage, whereas the extent of the nonlinear behavior decreased with increasing filler volume fraction. Zabihi et al. [109] constructed a Monte Carlo model of graphene/PMMA polymer by modeling graphene as a disk and combining it with the Landauer–Büttiker resistance formula. The study of the model revealed that the tunneling resistance between graphene and the metal is several orders of magnitude larger than the intrinsic resistance. The percolation threshold decreased with the increase in the tunneling distance and increasing graphene diameter. The dispersion of graphene perpendicular to the direction of the electric field causes the polymer to exhibit the smallest conductivity and the largest percolation threshold. In addition, Fang et al. [110] proposed a Monte Carlo model to calculate the conductivity of graphene-filled polymers using the equipotential approximation. The graphene model has coated surfaces (CSs) (Figure 5c), and the potential of the CSs can be derived from the graphene potential using the walk-on-spheres (WoS) method to obtain the potentials of all of the linked graphene, and it was demonstrated that the conductivity and percolation thresholds obtained by this model are in good agreement with the experimental data.

**Figure 6:**
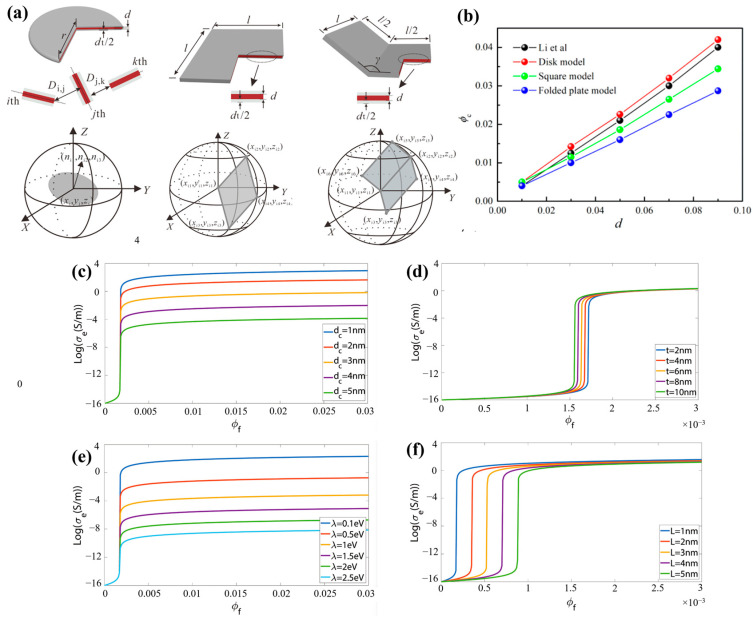
(**a**) Schematic diagrams of the disk model, square model, and folded plate model of graphene; (**b**) model predictions and experimental percolation threshold results [111]; (**c**–**f**) effective conductivity versus volume fraction for different parameters; (**c**) tunneling distance dc; (**d**) interface thickness; (**e**) polymer barrier; (**f**) graphene thickness [113].

Presently, most of the conductive material models in the research are generated by the “rand” function, but there exists the problem of the uneven probability distribution of the model orientation, which affects the position and normal vector of the conductive material model. Liu et al. [111] proposed the “uniform_real_distribution” function in C++ to generate the decimals uniformly distributed between 0 and 1, thus obtaining uniform polar and azimuthal angles for the graphene model. Thus, uniform polar and azimuthal angles are obtained for the graphene model, and the 3D Monte Carlo model of the graphene filled polymer is constructed on the basis of this function. Figure 6a,b show that the researcher compared the applicability of the disk model, the square model, and the folded plate model of graphene in this simulation by means of this model. The results show that the permeation volume fractions of the square and folded plate models were smaller than that of the disk model for the same parameters. The rectangular plate and the folded plate models can simulate the distribution state of graphene in the polymer more accurately, which leads to a more accurate prediction result. Wang et al. [112] modeled graphene as a rectangular plate with a hard core and established a Monte Carlo model for graphene-filled polymethylmethacrylate (PMMA). Through the study of the model parameters, it was found that the contact resistance between the graphene plays a decisive role in the overall resistivity of the polymer and that the smaller the size of the graphene, the better the electrical conductivity of the composite. A study by Payandehpeyman et al. [113] found that the model predicts the experimental results better for a polymer interface thicknesses of 1–2 nm and tunneling distances of 2–4 nm. Additionally, the decrease in the polymer potential barrier and the increase in the graphene aspect ratio had a positive effect on the electrical tunneling behavior, as shown in Figure 6c–f.

To study the feasibility of graphene–polypyrene (graphene-PPy) composites in the field of energy storage batteries, Folorunso et al. [114] combined Simmons and McCullough equations with Monte Carlo simulations to develop a numerical model for an in-depth study of the electrical properties of graphene-PPy composites. The study led to the following conclusions: tunneling resistance was a key factor in determining the electron transport in the composites; the tunneling distance of graphene in the polymer was strongly related to its diameter and the insulating thickness; and an increase in the length of graphene and a decrease in the insulating thickness led to a decrease in the tunneling distance, which, in turn, favors the electron transport.

### 3.4. Monte Carlo Models of Hybrid-Material-Filled Polymers

As carbon-based materials are prone to agglomeration, conductive networks are often formed in polymer matrices by increasing the amount of material added, excessive conductive materials will lead to increased costs and difficulties in processing the polymer and impairment of its own properties. Therefore, the percolation threshold for carbon-based materials in polymers. The simultaneous incorporation of conductive fillers with different morphologies into polymers has been shown to be an effective way to reduce the percolation threshold [115]. However, the experimental trend for the effect of hybrid fillers on the electrical properties of polymers is not clear, and there are two main trends in their electrical properties: additive effect [116,117] and synergistic effect [118,119]. The percolation threshold of the hybrid filler is lower than that of one of the fillers but higher than that of the other filler when an additive effect occurs. Synergistic effects occur when the percolation threshold of the hybrid filler is lower than that of any single filler. The synergistic effect is preferred over the additive effect for polymers filled with hybrid fillers. Experimentally exploring optimal hybrid ratios to produce synergistic effects is simple but laborious. Therefore, researchers have developed a variety of Monte Carlo models of hybrid materials filled polymers to analyze their synergistic conductive effects at the microscopic level. In this section, a systematic summary of recent Monte Carlo simulation studies on hybrid materials filled polymers is presented.

**Figure 7:**
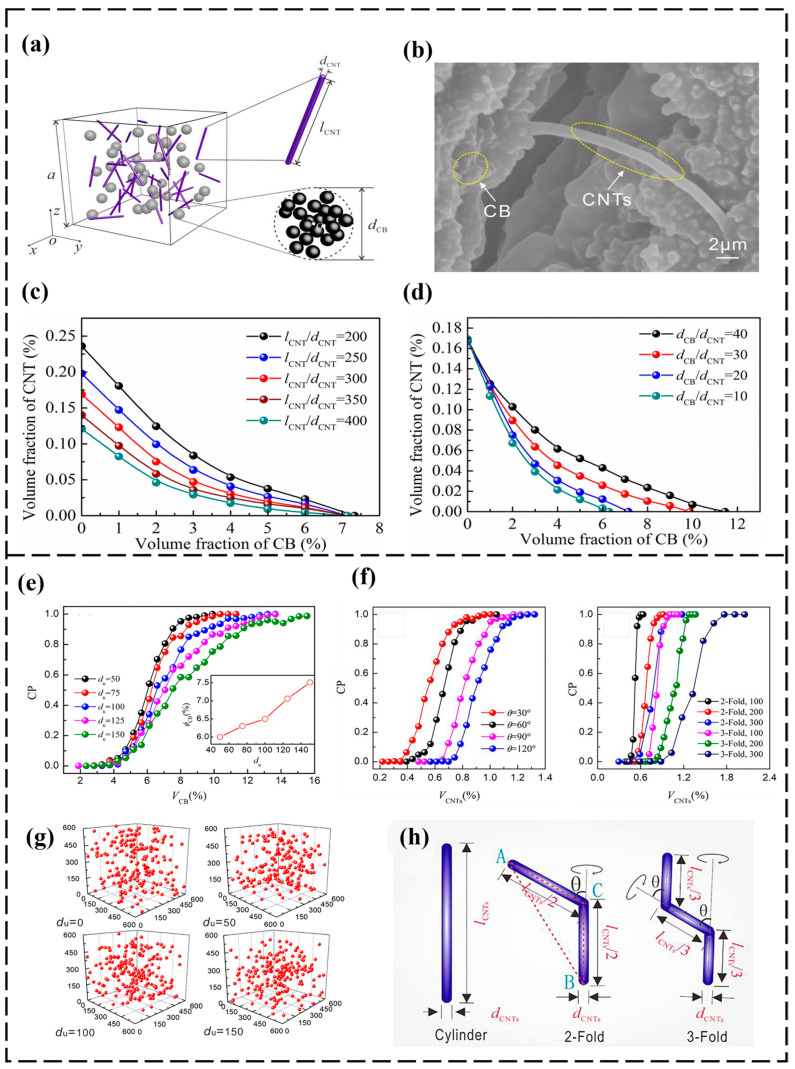
(**a**) Schematic of CB and CNT model filled RVE, (**b**) SEM images of CB and CNTs in polymer, (**c**) Dependence of synergistic effect on the L/D ratio of CNTs, (**d**) Dependence of synergistic effect on CB to CNTs’ diameter ratio [122], (**e**) Effect of the degree of CB agglomeration on the percolation behavior, (**f**) Distribution of CB in the RVE when the degree of agglomeration is set to 0, 50, 100 and 150, (**g**) Effect of fold angle and number of fold segments on polymer percolation behavior, (**h**) Schematic diagrams of rod and folded CNTs [124].

CNT materials have a rod structure with a large aspect ratio, making it easier to produce synergistic effects with other structures of conductive fillers. Therefore, they are mostly chosen as one of the hybrid components in the experiments and simulations of hybrid fillers. Conductive CB exists in the form of cluster aggregates in the polymer matrix, and it has better flexibility to intersperse and lap between CNTs, thus producing a synergistic conductive effect. Chen et al. [120] investigated the effects of the volume fraction and size of CB and CNTs on the electrically conductive percolation behavior of hybrid filler polymers by Monte Carlo simulation, which revealed a nonlinear relationship between the volume fractions of CB and CNTs at the hybrid percolation threshold, and it was consistent with the synergistic effect observed in the experiments. On the basis of the nonlinear relationship, an estimation equation was established for the electrical percolation threshold of the hybrid filler containing CB and CNTs. Yang et al. [121] combined the Monte Carlo method with the finite element method (FEM) to develop a model for the hybrid filling of CB and CNTs with PMMA. The model predicted that the filler fractions for the synergistic effects produced by CB and CNTs were 8% and 1%, which was a large deviation from the experimentally derived combinations of 5% and 2%. Also, the combination with the FEM makes the model more complex. Huang et al. [51,122] abstracted CBs and CNTs as regular spheres and cylinders with end caps and then constructed Monte Carlo models in Matlab 2017A software using a conductive path search algorithm developed on the basis of the KD-Tree data structure and graph theory, as shown in Figure 7a. For the CB and CNTs hybrid filling system, the conductive filler plays different roles in the formation of the conductive pathway, it can be seen in Figure 7b that the CB provides short-range connection, while the CNTs play the role of long-range bridging, and the two form a “bunch of grapes” structure, which leads to the existence of synergistic effects. The study found that the L/D ratio of CNTs and the diameter of CB are important factors affecting this synergistic effect, and the larger the L/D ratio of CNTs and the smaller the diameter of CB, the stronger the synergistic effect (Figure 7c–d). Ren et al. [123] came to a similar conclusion by studying a Monte Carlo simulation of the mixing of rod and spherical fillers. Lu et al. [124] quantitatively researched the electrical percolation threshold of hybrid SR compounds filled with CB and CNTs based on the study of Huang [122], and the results show that the simulation parameters of CB and CNTs can be optimized based on the experiments, where the degree of agglomeration of CB and the shape of the CNT model are the most important factors in the simulation (Figure 7e–h). Haghgoo [125] developed a Monte Carlo model predict to conductivity and percolation threshold of the hybrid CB and CNTs systems, and the model predictions were in good agreement with the experimental data. Furthermore, the effect of the polymer potential barrier on the percolation behavior was investigated by the model; the percolation threshold decreases with an increase in the barrier height.

**Figure 8:**
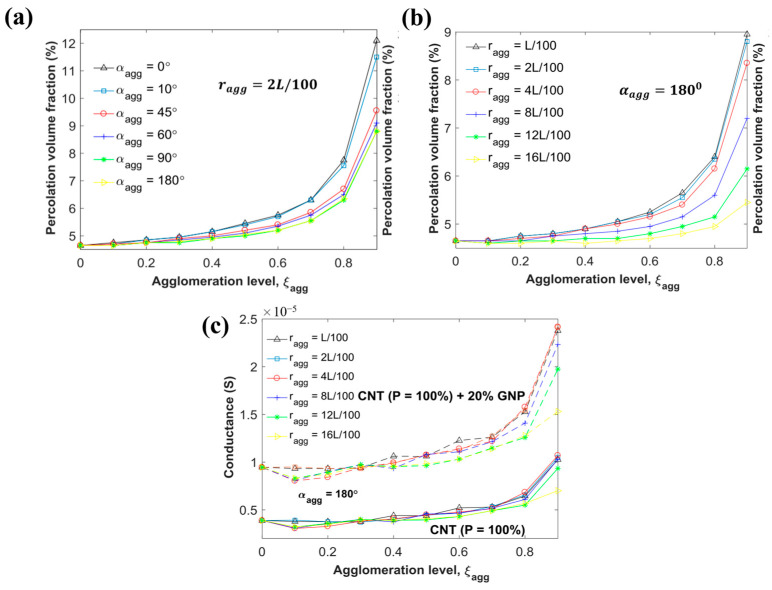
(**a**) Effect of agglomeration radius of CNTs on percolation threshold, (**b**) Effect of agglomeration angle of CNTs on percolation threshold, (**c**) Effect of addition of graphene on electrical conductivity of CNT-filled CPCs in agglomerated state [127].

The graphene material has excellent electrical conductivity and its percolation threshold in polymers is close to that of CNTs, while its plank-shaped structure with a high aspect ratio is favorable for the synergistic electrical conductivity effect with CNTs. At present, Monte Carlo simulations of graphene and CNT hybrid filled polymers have been reported in articles. Safdari et al. [126] used 3D Monte Carlo modeling to investigate hybrid polymer composites with graphene as the main conductor and CNTs as the auxiliary conductors. The addition of a small amount of CNTs to graphene significantly reduces the percolation threshold of the filled polymer system, and this enhancement of electrical conductivity is related to the relative aspect ratios (LAR) of graphene and CNTs, and adding a small amount of a high LAR auxiliary phase to a low aspect ratio main phase can improve the electrical conductivity of the composites by several orders of magnitude. A two-dimensional Monte Carlo model constructed by Gbaguidi et al. [127] investigated the effect of CNTs’ agglomeration on electrical percolation and conductivity. The model shows that a high degree of CNT agglomeration leads to an increase in the percolation threshold, and a decrease in the agglomeration radius and an increase in the agglomeration angle of the CNTs contribute to a decrease in the percolation threshold (Figure 8a,b). The incorporation of graphene as a cofactor phase results in an increase in electrical conductivity in polymer composites (Figure 8c). The above models of hybrid graphene and CNTs fillers only studied the additive effect, Gbaguidi et al. [128] constructed a 3D Monte Carlo model to study the synergistic effect of graphene and CNTs mixtures. It was found that when the percolation thresholds of graphene alone and CNTs alone in the filler were close to each other, the hybrid filler consisting of the two achieved a synergistic improvement of the electrical properties in the polymer. When the aspect ratio of graphene and the L/D ratio of CNTs are simultaneously increased, the hybrid percolation threshold of the polymer is lowered and the electrical conductivity of the polymer is increased. Wu et al. [129] implemented 3D Monte Carlo simulation in c++ environment found a similar phenomenon to Gbaguidi [128], i.e., when CNTs and graphene individually filled polymers have similar percolation thresholds and the length of CNTs is less than the diameter of the graphene, the polymers filled with the hybrid fillers are more likely to exhibit synergistic conductive effects in terms of electrical properties. Furthermore, Sohan et al. [130] found that the synergistic hybrid effect is directly related to the good dispersion of the fillers in the polymer and the tunneling interaction between the fillers.

From the above summary, we know that all the existing studies have used CNTs as one of the components, which were hybridized with CB and graphene, respectively; also, there are few reports on the simulation studies of CB and graphene blends as well as three filler blends filled with polymers; therefore, subsequent simulation studies on the synergistic conductive effects of CB and graphene hybrids as well as CB, graphene and CNTs hybrid filled polymers are needed.

## 4. Monte Carlo Models for Special Structured Polymers

The previous section presented Monte Carlo simulations of the electrical percolation behavior of carbon-based materials alone and of hybrid filled polymers, all of which are conventional static models. In recent years, researchers have built on conventional static models to construct special structural models in combination with flexible piezoresistive pressure sensors and foamed polymer materials, and the progress of such models is summarized in this section.

### 4.1. Monte Carlo Models of Polymer Piezoresistive Properties

Flexible piezoresistive pressure sensors have the advantages of a simple structure, high sensitivity, fast response, low manufacturing cost and good stability and are considered ideal for the next generation of flexible pressure sensors [131,132,133]. Filled CPCs are commonly used to prepare flexible piezoresistive strain sensors. Through the Monte Carlo model of the polymer piezoresistive effect, the structural distribution of the polymer conductive network and stress analysis can be analyzed, and it can provide an in-depth investigation of the microstructure of the sensor and the sensing mechanism, which can provide theoretical support for the design of sensors and the optimization of their performance [134]. In this section, we summarize the research progress in this field in an overview depending on the shape and structure of the conductive filler.

**Figure 9:**
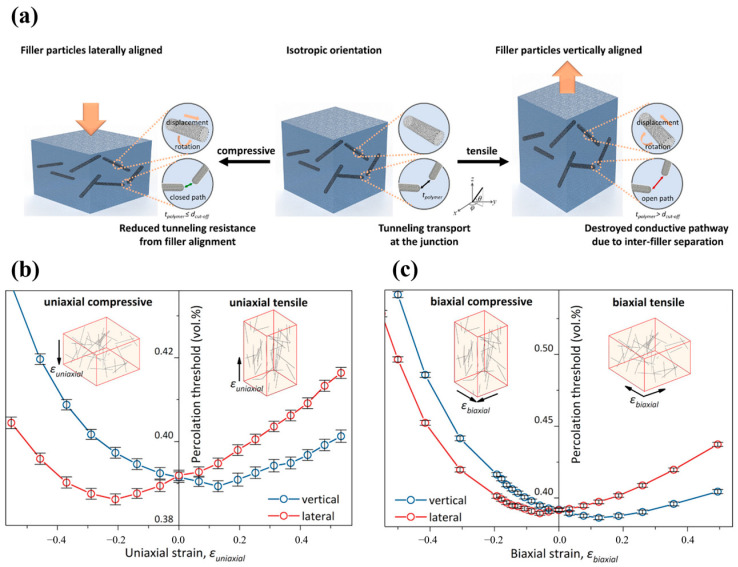
(**a**) Schematic representation of the structural response of CNTs in polymers after mechanical deformation; (**b**) effect of uniaxial strain on the vertical and lateral percolation thresholds; (**c**) effect of biaxial strain on vertical and lateral percolation thresholds [136].

Wang et al. [135] introduced the average junction gap variation (AJGV) into the model to quantitatively describe the strain-induced changes in the conductive network of CNTs, and found that AJGV plays a dominant role in the changes in polymer conductivity. The results of the simulation and analysis show that the AJGV is positively correlated with the changes in the diameter D and orientation angle θ of the CNTs, and negatively correlated with the Poisson’s ratio υ of the polymers; the AJGV increases and then decreases with the increase in the L/D ratio AR of the CNTs and finally tends to be stable. Meanwhile, this study gives the optimal design principle for the piezoresistive properties of CNT-filled CPCs, i.e., selecting larger diameter CNTs and smaller Poisson’s ratio polymers, while control the concentration of CNTs to a low level. Chang et al. [136] carried out theoretical modeling and experimental validation of the structural evolution of conductive polymers under mechanically deformed (uniaxial and biaxial compressive and tensile strains) (Figure 9a). In Figure 9b,c, the strain-induced packing arrangement changes the vertical and lateral percolation thresholds, with the vertical percolation threshold initially reaching a minimum and then gradually increasing as the uniaxial tensile strain (or equivalent biaxial compressive strain) in the vertical direction increases, while the lateral percolation threshold monotonically increases. On the other hand, with increasing uniaxial compression (or equivalent biaxial tension), the lateral percolation threshold reaches a minimum value and then gradually increases, while the vertical percolation threshold monotonically increases. The validity of the model was verified by comparing the piezoresistive predictions of PP-MWCNT nanocomposites, with experimental observations. Haghgoo et al. [137] analyzed the effect of the temperature effect on the piezoresistive properties of the polymers. The results showed that the piezoelectric conductivity of the polymers increased with an increase in temperature. In addition, the increase in the parameters such as the volume fraction of the CNTs, tunneling distance, uniformity of dispersion, and the polymer’s Poisson’s ratio had a significant effect on the decrease in piezoresistive susceptibility. The above is a representative study of the Monte Carlo method for simulating the piezoresistive properties of individual CNT-filled polymers.

**Figure 10:**
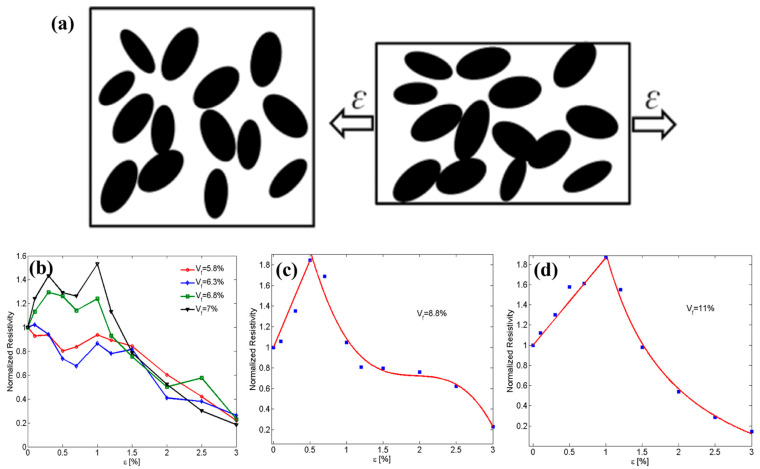
(**a**) Schematic of a graphene-filled polymer system subjected to tensile strain; (**b**) unstable piezoresistivity at a low filler volume fraction; (**c**) piezoresistivity at a 7.5% graphene volume fraction; (**d**) piezoresistivity at an 8.8% graphene volume fraction [138].

For the piezoresistive modeling of graphene-filled polymers, Oskouyi et al. [138] determined the new position and orientation of graphene in the deformed matrix (for uniaxial tensile strains) using affine transformations in their model, assuming that the polymers are subjected to mechanical strains by neglecting the folding and curving deformation of graphene (Figure 10a). In this research, Monte Carlo simulation computational code using the FORTRAN language was developed and ran on a Linux cluster. The volume fraction of filler was found to be an important factor affecting the piezoresistive behavior, and the polymer’s piezoresistive behavior was unstable at low graphene volume fractions. The polymer’s piezoresistivity was approximately linearly correlated with the strain as the graphene volume fraction increased until the critical strain, at which the resistivity behavior changed and the resistivity exhibited a monotonically decreasing trend as the strain continued to increase. Furthermore, the critical strain value increased with the increase in the filler volume fraction (Figure 10b–d). Yang et al. [139] found that the piezoresistive properties of graphene–rubber composites had a linear response at the initial stage of the strain and exhibited nonlinear characteristics as the strain increased. The model calculated the piezoresistive response of compounds containing 2.3% and 3.0% (wt.%) graphene, which is in good agreement with the experimental results.

Because the hybridization of conductive materials with different structures can produce a synergistic conductive effect, to further enhance the piezoresistive performance of polymers, scholars have used Monte Carlo models to investigate the effect of hybrid fillers on the piezoresistive performance of polymers. Haghgoo et al. [140] conducted 3D Monte Carlo simulations and studied the piezoresistive performance of polymer nanocomposites filled with a mixture of graphene and CNTs. The results showed that the length of the CNTs, orientation angle, edge length of the graphene, and polymer potential barrier height were positively correlated with the piezoresistive sensitivity. The volume fractions of the CNTs and graphene, as well as the Poisson’s ratio of the polymer, are negatively correlated with the piezoresistive sensitivity. In the same year, the team [141] investigated the effect of CB and CNT hybrid fillers on the polymer piezoresistive performance and found that shorter length CNTs, larger diameter CB particles, and a lower Poisson’s ratio were favorable for improving the sensitivity of polymer piezoresistivity.

In addition to the studies presented above, there are many other studies reported for this field in the literature, which are briefly summarized in Table 4. Currently, there are relatively few studies on the simulation of piezoresistive properties of graphene, as well as hybrid-material-filled polymers. Meanwhile, there are still some shortcomings in existing simulation studies; for example, the agglomeration and deformation of the conductive materials are not taken into account in the models, which limits the applicability of the models, especially for the relatively high-volume fraction of conductive materials.

### 4.2. Monte Carlo Models of Foamed Structured Polymers

Filled CPCs play an increasingly important role in all aspects of human society, but with the development of human society and the advancement of science and technology, higher requirements, such as low weight, high elasticity, and flexibility, have been put forward for filled conductive composites. By introducing foam structures into polymers, it is possible to reduce the weight of the polymers and improve the deformation capacity. However, the optimal effect of experimental samples is often difficult to obtain directly and needs to be explored through complex gradient tests, resulting in a large waste of time, manpower, and material resources. Through 3D Monte Carlo simulation, we can predict the results of the experiments to a certain extent before the experiments start and reduce the blindness of the experimental work.

**Figure 11:**
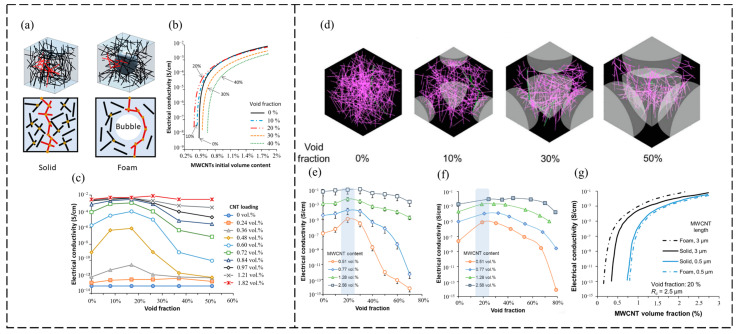
(**a**) Monte Carlo model of foamed polymer and schematic diagram of its conductive pathways; (**b**) variation in the conductivity with the volume fraction of the CNTs at different void fractions; (**c**) variation in the conductivity with the void fraction for the different CNT additions [149]; (**d**) simulated images of CNT networks in polymer foams at 0%, 10%, 30%, and 50% void fractions; (**e**,**f**) conductivity results for different volume fractions of CNT-filled foamed polymers at different void fractions; (**e**) model predictions; (**f**) experiments; (**g**) effect of CNT length on the conductivity of foamed polymers with a constant porosity, β, and bubble radius, Rc [150].

Shaayegan et al. [148] calculated the percolation threshold of polymer nanocomposite foams at various porosities with the help of Euler–Rodrigues rotation and Monte Carlo simulation; however, the model only uses a single bubble in the percolation simulation, and does not take into account the effect of multiple generated bubbles on the movement of the filler. Furthermore, the model uses a simplified percolation theory to calculate conductivity and does not distinguish between the mechanisms responsible for conducting electricity. Wang et al. [149] used the Euler–-Rodrigues rotation method and a modified Monte Carlo model to simulate the percolation threshold of foamed polymer composites, as shown in Figure 11a–c. The experimental and simulation results show that foaming can cause the alignment of filler particles around the bubbles, and the tiny alignment of filler particles can help to reduce the overall percolation threshold of polymer foams containing rod fillers, and at a void fraction of less than 20%, the conductivity of the polymer decreased. The optimal void ratio was approximately 17%, at which point the percolation threshold of the foam specimen decreased by 20%, from 0.6 vol% to 0.48 vol%. Further bubble growth can reduce filler particle interconnections, thereby disrupting the conductive network. On this basis, the team [150] also considered the simultaneous growth of multiple bubbles in the polymer (Figure 11d). Because the model did not consider the agglomeration of CNTs, the predicted conductivity was higher than the experimental data when the content of CNTs exceeded 1.28 vol% (Figure 11e,f). Also, when the bubble size is comparable to the length of the CNTs, there is an opportunity to rearrange and localize the CNTs to an optimal extent (Figure 11g), thereby improving the electrical properties of the foamed CPCs. Peng et al. [151] performed simulations using Ansys software and found that in the single filler model, the radius of the spherical filler and the radius of the bubble decreased, the aspect ratio of the rod filler, and the fraction of voids in the system increased, which promoted the conductive percolation behavior of the system. In the sphere-rod hybrid filler system, when the radius of the sphere filler was about two times that of the rod filler, the synchronicity of the two fillers was better and the conductivity of the system was optimal.

Monte Carlo models serve as a useful predictive tool for the design and optimization of carbon-based fillings in foam CPCs. However, few studies are available, so there is still great potential for research in this area.

## 5. Summary and Perspectives

CPCs with carbon-based fillings have a wide range of applications in many fields, and various aspects of carbon-based fillings in CPCs have been synthesized by scholars in earlier published works; however, the simulation of the electroosmotic flow of carbon-based fillings in CPCs has not yet been synthesized. In this paper, we provide a comprehensive summary of the Monte Carlo method, which is the most commonly used method for the electroosmotic simulation of CPCs with carbon-based fillings.

First, we summarized the construction mechanism of the conductive percolation model, including conductive channel theory, tunneling effect theory, and field-emission effect theory, with different triggering conditions. Although occupying the role of the dominant conductive mechanism is different, the three kinds of conductive mechanisms were used together in the model for the realization of the phenomenon of electrical percolation to provide a theoretical basis. The Monte Carlo method is able to predict the distribution of carbon-based fillers in a polymer system and the electric percolation behavior, meanwhile, it can research and analyze the factors affecting the electric percolation behavior from a microscopic point of view. In the second part of the thesis, on the basis of the difference in the physical structure of carbon-based materials, detailed Monte Carlo simulation studies on the filling of polymers with CNTs, CB, graphene, and hybrid materials were summarized. Currently, the Monte Carlo models of CNT-filled CPCs are very mature, and it was found that the 3D RVE space and the folded and curved models more realistically responded to the shape, structure, and spatial distribution of the CNTs inside the polymers. Using the model, the effects of CNTs’ L/D ratio, chirality, length distribution, and aggregation, as well as the potential barrier of the polymers on the electric percolation behavior, can be analyzed, and this can guide the actual production and experiments with conductive polymers.

Similarly, to the CNT-filled CPC model, the study of the shape and structure of graphene in the model revealed that the rectangular plate and folded plate models can more accurately simulate the distribution of graphene in polymers than the disk model, resulting in more accurate predictions. In addition, researchers have also reported the effect of factors such as the aspect ratio and size of the graphene on the electrical percolation behavior and some valuable conclusions have been obtained. However, the models established in most of the current studies are static simulation models without the influence of external factors, while the main application scenarios of graphene-filled CPCs (flexible sensors, electronic skins, etc.) do not match, so it is necessary to introduce the functional relationship between the temperature, humidity, applied stress–strain, etc., and the percolation thresholds in order to analyze the dynamic changes in the percolation thresholds under the influence of the external environment.

Compared with CNTs and graphene, the Monte Carlo simulation of CB-filled CPCs is less studied, because CB itself is generally conductive and does not have a large aspect ratio structure, which requires a large amount of addition to the polymer to achieve the desired resistance, which will lead to a significant reduction in the performance of the polymer itself. Monte Carlo simulation of CB-filled polymers can be applied to the microstructural design of filled conductive polymers, such as the combination of CB with a large aspect ratio and high conductivity fillers such as CNTs and graphene.

The percolation behavior and the synergistic conductive phenomenon of hybrid filler in polymer systems are the research hotspots of Monte Carlo simulation in recent years, and through the summary of the literature, the previous research often takes CNTs as one of the components, respectively, and CB and graphene hybrid to construct the model. Researchers found that the larger the aspect ratio of CNTs and the smaller the diameter of CB, the stronger the synergistic effect. Graphene and CNTs that individually filled polymers have similar percolation thresholds, and when the length of CNTs is smaller than the diameter of graphene, the hybrid exhibits a better synergistic conductive effect. Currently, there are no reports in the literature on studies simulating a CB and graphene hybrid; three kinds of conductive filler hybrids; and the electrical percolation behavior of hybrid materials filled CPCs, and this has not yet formed a complete system.

In addition, this paper summarizes the progress of research on dynamic piezoresistive models, as well as polymer models with foamed structures. Research on dynamic piezoresistive modeling has focused on single CNT-filled polymer systems. The reported models are capable of predicting the piezoresistive properties of polymer composites and analyzing factors that affect the piezoresistive properties, which provide a basis for the design of flexible piezoresistive pressure sensors. For the foamed structural polymer model, it was found that the foaming effect induces the alignment of the filler particles around the bubbles, and the tiny alignment of the filler particles helps to lower the overall polymer percolation threshold, but has a negative effect on the electrical percolation behavior as the pore size of the bubbles increases and the porosity increases. Although some valuable conclusions have been drawn from research in these two areas, more research is needed to validate these conclusions due to the paucity of available literature.

Over the past two decades, researchers have used the Monte Carlo method to simulate the electrical percolation behavior of different carbon-based fillings in CPCs and to analyze the factors affecting the percolation behavior of the polymers from a microscopic point of view, which provides ideas and a basis for the design of filled CPCs with better performance. However, research on the structural design of filled CPCs is not only limited to static single polymer systems; in addition to the piezoresistive polymers and foamed polymers introduced in the paper, there are also double percolation systems containing conductive polymers, among others. Additionally, there are still fewer studies on the integration of this simulation with practical applications, and the Monte Carlo simulation of CPCs filled with different kinds of carbon-based materials should be increased to combine with electromagnetic interference shielding, antistatic, flexible sensors, and wearable electronic devices, so as to design CPCs with a more comprehensive performance and that are closer to practical production applications.

## Figures and Tables

**Table 1:** Structure and properties of carbon-based materials.

Materials	Physical Structure	Density (g/cm^−3^)	Conductivity (s/cm)
Carbon nanotubes (CNTs) [17]	Rod	1.3–1.75	10^3–^10^4^
Carbon fiber (CNF) [17]	Rod	2	10^4^
Graphene [18]	Plate or disk	1.06	10^4^
Carbon black (CB) [19]	Sphere	1.8–2.1	0.1–10

**Table 2:** Summary of Monte Carlo simulation studies of CPCs filled with CNTs.

RVE	CNTs’ Structure Parameters	Influencing Factors or Research Content	Important Results	Ref. and Years
2D	Rod, soft-core model.	CNTs’ polydispersity L/D ratio.	Prediction by the polydispersity aspect ratio models closer to experimental results.	Arenhart [69] 2016
	Fold model.	Electromagnetic shielding effectiveness (SE) of polymers.	Use of the simulated conductivity data to calculate the SE, with the results agreeing with both the experimental and theoretical values.	Prabhu [70] 2017
	Rod model.	Electrical conductivity of CNT-filled films studied with continuum and lattice models.	Both models exhibited similar behavior, leading to a dependence of the conductivity on the rod concentration in low concentrations of rods.	Tarasevich [71] 2018
	Rod model, length followed Weibull distribution.	Tunneling resistance. CNTs’ length distribution.	The effect of tunneling is significant in CNTs with small aspect ratios. The length distribution of CNTs yields a smaller percolation threshold.	Doh [72] 2019
3D	Rod model.	Tunneling resistance.	Tunneling resistance dominates the conductivity of polymers.	Li [73] 2007
	Rod model and curved model, length followed normal distribution.	CNT shape model. CNTs’ aggregation.	The curved CNT model has a high percolation threshold and low electrical conductivity. The aggregation of the CNTs raises the percolation threshold.	Hu [74] 2008
	Two-segment fold model.	CNTs’ folding angle. CNTs’ L/D ratio.	The percolation threshold decreased with the increase in the L/D ratio of the CNTs. The CNTs’ L/D ratio was small, and the folding angle had a large effect on the percolation behavior. The percolation threshold decreased with a decrease in the folding angle.	Ma [75] 2008
	Ten-segment fold model.	Van der Waals interactions between CNTs. Tunneling effects. CNTs’ L/D ratio. CNTs’ folding angle.	The influence of van der Waals and tunneling effects on the percolation behavior of overdrafts diminishes with an increase in the L/D ratio. The percolation threshold becomes larger with the increase in the maximum folding angle of CNTs.	Lu [76] 2010
	Rod model, length followed Weibull distribution.	CNTs’ orientation.	The conductivity was highest when CNTs are partially aligned rather than isotropic. As the concentration of the CNTs decreased, the optimal orientation tended to be isotropic.	Bao [77] 2011
	Rod model, length followed Weibull distribution.	CNTs’ agglomeration.	Agglomeration enhanced the electrical conductivity of the polymers with a lower CNT content, while the effect with a higher content was insignificant.	Bao [78] 2012
	Ball chain structure, ball size (1–100 nm), ball chain number (16–64).	CNTs’ flexibility. CNTs size dispersion. Molecular attraction.	The percolation threshold increased significantly with an increase in the size dispersion or flexibility. Rigid CNTs have strong mutual attraction and easily agglomerate.	Lee [79] 2012
	Fold model, length followed Weibull distribution.	Polymer potential barrier. CNTs’ shape. CNTs’ length. CNTs’ folding angle.	At the percolation threshold, the length distribution and folding angle have a greater influence on the percolation behavior. Past the threshold, barrier height has a greater influence on the percolation behavior.	Bao [80] 2013
	Rod model.	Use of Monte Carlo method to design a micromechanical model.	Calculated effective conductivities of uniformly distributed, randomly oriented CNTs filled with epoxy resin.	Kulakov [81] 2017
	Several carbon atoms used build a rod model.	Effect of CNTs’ cross angle on the contact resistance of CNTs.	The CNTs’ contact resistance is more dependent on the cross angle when the cross angle spans from 0 to π/2.	Khromov [82] 2017
	Rod model, length followed normal distribution.	Infinite cluster structure in the region of percolation transition.	The study determined the order of the parameters and the functional form of the conductivity close to the percolation transition.	Gennadiy [83] 2018
	Hollow curved structural model.	Combined FEM calculations to predict electrical properties of CNT-filled polymers.	The model can be implemented in Abaqus, is able to capture tunneling conductivity effects at the junction of adjacent CNTs, and does not use fitted parameters to calibrate against experimental results.	Matos [84] 2018
	Curved model.	CNTs’ length and diameter and polymer interface thickness on the effective volume fraction of CNTs.	CNTs’ effective volume fraction increased with a decreasing CNT radius and increasing filler concentration and interfacial thickness. CNTs’ length had little effect on the effective volume fraction.	Zare [85] 2019
	Rod model.	CNTs’ L/D ratio. CNTs’ diameter and chirality. Polymer potential barrier.	The percolation threshold decreased with a decrease in the potential barrier. Higher aspect ratios of the CNTs led to lower thresholds. Below the threshold, polymer conductivity was not affected by the diameter and chirality.	Fang [86] 2019
	Rod model.	CNTs’ orientation.	CNTs’ orientation changed from isotropic to anisotropic, and both the percolation threshold and resistivity decreased.	Dong [87] 2020
	Curved model.	CNTs’ L/D ratio. CNTs’ waviness. CNTs’ volume fraction. CNTs’ orientation.	CNTs’ L/D ratio and waviness degree increased the conductivity, and this effect was more prominent in an isotropic orientation. CNTs’ volume fraction increased, and the difference between the isotropic and anisotropic conductivities of the CNTs gradually decreased.	Chanda [88] 2021
	Several carbon atoms used to build a rod model.	CNTs’ cross angle.	On the basis of the molecular dynamics and Monte Carlo method, a multiscale model of CNTs populated with R-BAPB was constructed. It was found that the contact resistance of the CNTs was dependent on the cross angle.	Larin [89] 2021

**Table 3:** Comparison of the speed and number of nodes accessed by different algorithms [98].

Algorithm	Composite Type	Filler Diameter (nm)	Visited Node Count	Average Time to Find Path
A* Algorithm	TPU/CB	10	32	2.444
A* Depth Algorithm	TPU/CB	10	35	2.869
Best-First Search	TPU/CB	10	25	2.288
Breadth-First Search	TPU/CB	10	2090	185.507
Dijkstra’s Algorithm	TPU/CB	10	577	44.363
Fastest Path Search	TPU/CB	10	44	4.218

**Table 4:** Summary of Monte Carlo modeling studies of polymer piezoresistive effects.

Materials	Research Content	Important Results	References	Years
CNTs	Tunneling effects and internal conductive network contributions to piezoresistive performance.	These two parameters play an important role in piezoresistivity.	Hu [142]	2012
	Morphology and intrinsic resistance of CNTs on piezoresistive properties.	The lower aspect ratio and intrinsic resistance of CNTs lead to higher piezoresistivity.	Gong [143]	2014
	Influence of interfacial and tunneling effects on piezoresistive properties.	Lower interfacial resistivity of the CNTs and higher effective stiffness of the polymers reduced the piezoresistive sensitivity.	Souri [144]	2017
	A combined FEM and Monte Carlo method to establish a coupled electromechanical multiscale model.	Predicted piezoresistive behavior of CNT-epoxy under tensile, compression, and shear loading is in good agreement with the experimental results of earlier studies.	Alian [145]	2019
CNTs, Graphene	Effect of the size of CNTs and graphene hybrid fillers on piezoresistive sensitivity.	The high aspect ratio/transverse size and filler specific surface area of the CNTs and graphene improved the piezoresistive sensitivity.	Avilés [146]	2018
	Effect of the degree of agglomeration of CNTs on piezoresistive properties.	Lower agglomeration of the CNTs enhances the piezoresistive performance. Doped graphene favors piezoresistive properties.	Gbaguidi [127]	2019
	Effect of filler size and polymer potential barrier on piezoresistive properties.	CNTs and graphene have a large and similar size and polymers have a high potential barrier, which favor piezoresistive properties.	Liu [147]	2022

## Data Availability

Not applicable.

## Abbreviations/Nomenclature

Electrical percolation behavior:	The behavior of a polymer that mutates from an insulating state to a conducting state with the addition of a conductive filler to the polymer.
Percolation threshold:	The critical filling fraction of a polymer when it changes from an insulating to a conducting state.
Monte Carlo method:	The use of random numbers (or more commonly pseudo-random numbers) to solve many computational problems.
Tunneling theory:	Tunneling is a quantum effect determined by the fluctuations of microscopic particles. Also known as potential barrier penetration.
Field emission theory:	The strong external environment enables the electrons inside the matrix to overcome the binding of the nucleus and move freely to form an electric current.
Tunnelling distance:	The maximum distance between conducting materials in a polymer that generates tunnelling current.
RVE:	Representative volume element refers to the range of averages selected for mathematical modeling of continuous media at the macroscopic level using the local volume averaging method.
Polymer barriers:	Refers to energy barriers in polymers that prevent electron transfer between conducting materials.
Polymer Poisson’s ratio:	The ratio of the absolute value of the positive transverse strain to the positive axial strain when the polymer is subjected to unidirectional tension or compression, also called the transverse deformation coefficient.
Double percolation theory:	The theory of double percolation proposes that the distribution of conductive fillers in incompatible polymers at one-phase or two-phase interfaces can greatly reduce the percolation threshold of CPCs and improve the processing performance and mechanical properties of CPCs.

## Appendix A

**Table A1:** Summary of Monte Carlo simulations and experimental results on the electrical percolation threshold of polymers filled with different carbon-based materials.

Materials	Polymer	Materials Parameter	Percolation Thresholds		References
			Simulation	Experimental	
CNTs	Thermoplastic	L/D = 140–240	1.75–3.25 vol. %	1.47–2.94 vol. %	[60]
	Epoxy	L = 1.5 μm	1–2 wt.%	1–2 wt.%	[61]
		L/D = 160			
		L = 5 μm	1.5–2 vol. %	1–1.5 vol. %	[63]
		L/D = 100			
		L = 430 nm	2–3 vol. %	1.8–2.5 vol. %	[64]
		L/D = 43			
		L = 5 μm	1.5–2 vol. %	1–1.5 vol. %	[65]
		L/D = 100			
		L = 5 μm	1 vol. %	1–1.5 vol. %	[77]
		L/D = 100			[78]
		L = 5 μm	0.7 vol. %	1–1.5 vol. %	[84]
		L/D = 100			
	PMMA	L = 1.5 μm	0.4–0.6 vol. %	0.5–0.8 vol. %	[68]
		L/D = 160			
	Polyamide (PA-6)	L = 6 μm	0.5–0.7 vol. %	0.5–0.7 vol. %	[68]
		L/D = 200			
	Polyimide	D = 1.018 nm	0.03–0.06 vol. %	0.05 vol. %	[86]
		L/D = 2000			
	Polypropylene (PP)	D = 2.584 nm	3–4 wt.%	3.8 wt.%	[86]
		L/D = 21			
CB	Polyurethane (PU)	D = 168 nm	6.2 wt.%	6 wt.%	[94]
	Polydimethylsiloxane (PDMS)	D = 324 nm	Consistent with experimental results	4.52 vol. %	[95]
		D = 288 nm		6.96 vol. %	
		D = 236 nm		7.76 vol. %	
	Thermoplastic Polyurethane (TPU)	D = 20 nm	5.6 wt.%	5.6 wt.%	[98]
	Polypropylene (PP)	D = 68 nm	13.5 vol. %	7.48 vol. %	[99]
	Polyethylene Terephthalate (PET)	D = 68 nm	11.1 vol. %	8.46 vol. %	
	Nylon	D = 68 nm	30.5 vol. %	27.38 vol. %	
	Castor-oil Polyurethane (PUR)	D = 35 nm	5.2 vol. %	5.7 vol. %	
	Nature Rubber (NR)	D = 15 nm	7.2 vol. %	7.3 vol. %	
Graphene	Polymethyl Methacrylate (PMMA)	D = 3 nm,5 nm,7 nm	2–3 vol. %	0.25 vol. %	[109]
		D = 5 μm, L = 0.9 nm	0.5 vol. %	0.6–0.8 vol. %	[113]
	Polyethylene Terephthalate (PET)	D = 45 μm, L = 1.57 nm	0.4–0.45 vol. %	0.47 vol. %	
	Polyurethane (PU)	L = 1 nm	0.03–0.05 vol. %	0.078 vol. %	
	Polymerized Cyclic Butylate Terephthalate (PCBT)	D = 5 μm, L = 6 nm	2–3 wt.%	4 wt.%	
	Polystyrene (PS)	L = 1 nm	0.1–0.15 vol. %	0.15–0.3 vol. %	
		D = 50 μm, L = 2 nm	1–1.5 vol. %	2–3 vol. %	[114]
